# Validation of Calcein Violet as a New Marker of Semen Membrane Integrity in Domestic Animals

**DOI:** 10.3390/ani13111874

**Published:** 2023-06-04

**Authors:** Sophie Egyptien, Benjamin Dewals, Fabien Ectors, Flore Brutinel, Jérôme Ponthier, Stéfan Deleuze

**Affiliations:** 1 Fundamental and Applied Research for Animals and Health Research Unit (FARAH), Comparative Veterinary Medicine, Faculty of Veterinary Medicine, University of Liège, Avenue de Cureghem 1, 4000 Liège, Belgiumjerome.ponthier@uliege.be (J.P.);; 2 FARAH, Veterinary Public Health, Faculty of Veterinary Medicine, University of Liège, Avenue de Cureghem 1, 4000 Liège, Belgium

**Keywords:** epifluorescence, flow cytometry, spermatozoa, viability, acrosome, mammals, insects, birds

## Abstract

**Simple Summary:**

One way to conserve biodiversity is to cryobank good quality semen. Unsurprisingly, analysing multiple parameters improves semen quality and fertility evaluation. Semen membrane integrity, which indicates sperm cell viability and acrosomal status that reflects sperm-oocyte fusion capacity, are some of the basic parameters evaluated, among many others. Specific fluorescent markers (fluorochromes) that emit mostly green and red light routinely assess these parameters. Color overlapping limits simultaneous evaluation of multiple parameters. Small volume ejaculates, which naturally occur in roosters, small dogs and honeybees, will preclude repetition of analysis, thus interfering with fertility estimation. The use of fluorescent semen quality markers emitting in different light channels is the most appropriate for multiple parameter analysis. The purpose of this experiment is to establish Calcein Violet, a blue fluorochrome, as a marker of viability and acrosomal status in domestic animals in order to free the red and green channels for concomitant analysis of other parameters. Our serial analyses validate Calcein Violet for evaluation of membrane integrity and acrosomal status. Its use in multiple parameters analysis should therefore be recommended.

**Abstract:**

Many fluorochromes routinely used in semen quality analysis emit in the green and red channels, limiting their possible combination for multiple parameter analysis. The use of fluorophores emitting in different light channels broadens the possibilities of combination to expand the range of simultaneously evaluated criteria. This is of great interest in cases of small ejaculated volumes, such as those naturally occurring in roosters, small dog breeds and drones (*Apis mellifera*). The purpose of this experiment is to establish Calcein Violet (CaV), a blue fluorochrome, as a marker of viability and acrosomal integrity in domestic animals in order to free the red and green channels. SYBR^®^14/Propidium Iodide (PI) was used as reference dye, heat-treated samples as negative controls, serial staining combination for validation and epifluorescence microscopy for observation. Dead spermatozoa marked in red with PI showed no blue fluorescence either from the head or the tail. Live spermatozoa showed a decreasing blue emission from head to tail when single stained with CaV. Unreacted acrosomes showed intense blue fluorescence irrespective of plasma membrane integrity. This needs to be further confirmed for species with small and difficult to observe heads. Establishment of CaV as a marker of membrane integrity by fluorescence microscopy is a decisive first step towards further technical development and use with flow cytometry.

## 1. Introduction

Fertility prognosis is of great interest for breeding decision and semen cryobanking in general, but most particularly in the context of biodiversity preservation. In vitro quality parameters of fresh and post-thaw semen of mammals are commonly used to predict fertility rates in veterinary medicine. Semen concentration, motility, morphology, viability, acrosomal status, peroxidation of membrane lipids and DNA fragmentation are among the evaluated criteria. Assessment of semen concentration, motility and viability is routinely performed either manually or with computer-assisted sperm analysis (CASA) systems. Additional parameters, such as acrosomal status, DNA fragmentation and lipid peroxidation, are further evaluated in cases of clinical infertility, in artificial insemination centers [1] and in research. This is usually achieved using specific fluorochromes, either by epifluorescence or by flow cytometry [2,3,4,5].

Semen evaluation must be run on multiple parameters to develop reliable predictive models of fertility [2,6]. An obvious limitation to simultaneous multiple parameters fluorescent analysis is spectrum overlapping. Unfortunately, most of the fluorochromes used for semen evaluation emit in the green and red light spectrum channels, limiting their combinations. Freeing these channels, at least for some criteria, immediately broadens the possibilities of combinations. Another strategy to increase the number of parameters evaluated is to multiply the number of analyses. This seems easy for species with large volume ejaculates but is self-limiting for species and donors with small ejaculated volumes. It also problematic in cases where of low numbers of ejaculates are likely, such as with wild animals, where repetition of semen collections can be challenging for obvious reasons. The only real game plan is to improve the efficiency of the analyses and minimalize their repetitions.

Spermatozoa viability, a core parameter, can be grossly assessed by their motility, which, in turn can be used as a predictive marker of fertility in domestic animals [7,8,9,10,11,12]. According to the World Health Organization, the percentage of live spermatozoa is assessed by identifying those with an intact cell membrane by dye exclusion (dead cells have damaged plasma membranes that allow for entry of membrane-impermeant stains) [13]. Membrane integrity status objectively evaluates viability and is routinely assessed using a double staining with SYBR^®^14 (SYBR) and propidium iodide (PI) [14]. Under adequate technical assembly, SYBR emits observable green fluorescence (Excitation peak 490 nm, Emission peak 520 nm) and PI red fluorescence (Excitation peak 304 nm, Emission peak 620 nm). SYBR readily permeates through the membrane of live cells and binds to nuclear double helix DNA, resulting in the labelling of all cells. PI is a large molecule that only enters dying or dead cells with damaged membranes, resulting in nuclear DNA binding and quenching of SYBR-emitted fluorescence signal. Thus, a SYBR/PI dual staining allows for the discrimination of viable (SYBR+ only) and membrane compromised or dead sperm (PI+) [13,14,15].

Calcein violet (CaV) is another fluorochrome that has been suggested as a potential candidate for semen evaluation [16], and has recently been used in bull semen to assess cell viability using flow cytometry [3,17]. The acetoxymethyl derivative of Calcein Violet (Calcein Violet 450 AM) is permeant and non-fluorescent. After hydrolyzation of Calcein Violet 450 AM by intracellular esterases into CaV, an impermeant viability dye, it can emit blue fluorescence (Excitation peak 400 nm, Emission peak 452 nm). Damaged membranes do not retain CaV, thus allowing discrimination of membrane integrity. As esterases are generally distributed in the cell [18,19], it may be expected that CaV should mark in blue the acrosome [20,21,22], the head and the tail of spermatozoa with intracellular esterase activity and an intact membrane [23].

Acrosomal integrity evaluation with peanut agglutinin (PNA) conjugated to a fluorescent marker fluorescein isothiocyanate (FITC-PNA) or phycoerythrin (PE-PNA) is validated for domestic mammals [24,25,26,27]. PNA binds to carbohydrate of glycoproteins and glycolipids of the outer acrosome membrane. PNA is membrane impermeable, and thus, in the absence of cell permeabilization, conjugated PNA marks acrosomes of spermatozoa with reacted acrosome or impaired sperm plasma membrane [28]. PE emits in the orange wavelength (Excitations peaks 496, 546 and 565 nm, Emission peak 578 nm) and FITC in green wavelength (Excitation peak 494 nm, Emission peak 517 nm).

The fact that CaV emits in shorter wavelength and is detected in the common blue channel used in microscopy or flow cytometry should be seen as an opportunity for multiple colour staining and avoid spectral overlapping. Thus, validating its use for the evaluation of semen viability would prove highly valuable as an alternative to commonly used dyes. 

The aim of this paper is to investigate, characterize and validate the use of CaV for viability assessment of semen and acrosome membrane integrity from different species of domestic animals (horses, bulls, dogs, boars, roosters and drones) using fluorescence microscopy, as it is the recommended method to validate new stains [29]. To the best of the authors’ knowledge, this is the first study to thoroughly validate the use of CaV using reference dyes and controls. Not only does our work establish CaV as a marker of spermatozoa membrane integrity in the six species studied, it also shows CaV to be a marker of acrosome membrane integrity in the four mammal species. This is a stepping stone towards the development of using CaV for semen evaluation in other species and with flow cytometry.

## 2. Materials and Methods

### 2.1. Semen Collection

For bull, horse, dog and boar, five individual semen samples were used: two for Experiment 1 and three for Experiment 2. Bull and stallion semen samples were obtained from frozen thawed straws. Straws were plunged in water at 37 °C for 30 s for thawing. Donors of the other species were all hosted at the Veterinary Faculty of the University of Liège (Liège, Belgium). Drone semen was collected from 100 individuals one month prior to analysis and kept in the dark after pooling in a sealed straw at 18 °C as commonly described [30]. Semen samples of roosters, dogs and boars were manually collected in the morning and stained early in the afternoon on the same day. The rooster semen samples were pooled after collecting from three animals.

### 2.2. Semen Evaluation

The same operator evaluated all samples. Semen concentration was evaluated using a haemocytometer (Thoma cell counting chamber, Marienfeld^®^, Germany) for all species with a solution of 10 µL of semen diluted in 390 µL of formaldehyde 2%. Heads of spermatozoa were counted in 10 squares of 0.2 mm under optical microscope at ×400. The concentration of the undiluted semen sample was calculated following the formula: (number of heads/0.04) × 40 × 1000 = concentration in millions of spermatozoa/mL.

For all samples, a drop of 10 µL of semen was deposited on a prewarmed slide and evaluated under brightfield optical microscopy equipped with a stage warmer set at 37 °C at a final magnification of ×400, and motility was evaluated prior to any semen manipulation. Percentage of massal and progressive motility was assessed by an experienced operator as described by the World Health Organization [13] in all species except for drones. Drone semen motility was described as motile if any movement was observed [31].

All samples were included regardless of semen quality and potential fertility.

### 2.3. Semen Dilution and Samples Preparation

To facilitate individual observation of spermatozoa under epifluorescence microscopy, raw semen was diluted to a final concentration of 1–10 × 10^6^ spermatozoa/mL [29]. Semen extenders used were species specific. For the stallion, INRA 96^®^ was used (IMV Technologies, L’Aigle, France) [32]. For dog [33] and cattle [34], homemade TRIS-fructose based diluent was used. For boar, Vitasem^®^ (Magapor, Zaragoza, Spain) was used [35]. For rooster, Lake and Ravie extender [36] was used and finally for drone semen, TES + TRIS based extender was used. Homemade TRIS-fructose based diluent [37] and drone semen extender TES + Tris based [38] were prepared as previously described.

#### 2.3.1. CaV Validation as a Marker of Viability (Experiment 1)

Each diluted semen sample was divided into two aliquots. One was not heat-treated and was kept at 37 °C for horses [32] and bull [34] semen, at 20 °C for dog [33], boar [6] and drone semen [30], and at for 4 °C for rooster semen [36]. The second one was heat-treated to prepare dead control cells. Heat-treated samples were processed as recommended by ThermoFisher Scientific LIVE/DEADTM Sperm Viability kit protocol [39]. Briefly, diluted semen in 1.5 mL aliquot was plunged into a hot water bath for 20 min at 60 °C and then left to cool down at room temperature. These samples were used as controls with a population of dead spermatozoa. After heat treatment and before staining, motility was assessed for the second time, as described earlier. The complete experiment was run on two replicates for all species.

#### 2.3.2. CaV Validation as a Marker of Acrosomal Membrane Integrity (Experiment 2)

This part of the experiment was run on species whose large sperm heads [40,41,42,43,44,45] allowed easy evaluation of the acrosome, i.e., the dog, bull, stallion and boar. Three different donors were used per species tested (three dogs, three bulls, three stallions and three boars) in order to run the analyses in triplicates. The diluted semen was kept non-heat-treated at 20 °C.

### 2.4. Fluorochromes and Epifluorescence Evaluation

#### 2.4.1. CaV Validation as a Marker of Viability (Experiment 1)

For all species, two samples (heat-treated or not) of diluted semen (1–10 × 10^6^/mL), were either triply stained (CaV + SYBR+ PI), double stained (CaV + PI) or single stained (CaV). Three controls were not heat-treated and were either single stained (SYBR or PI) or left unstained, as a negative control allowing for autofluorescence assessment.

Working solutions of the three fluorochromes were prepared as recommended by their manufacturer. A volume of 1.5 µL of a 20 µM SYBR^®^14 in DMSO solution (LIVE/DEADTM Sperm Viability kit, ThermoFisher Scientific, Waltham, MA, USA) was added to 50 µL of diluted semen and incubated in the dark at room temperature for 10 min. A volume of 4 µL of a 93 µM CaV in PBS solution (eBioscienceTM Calcein Violet 450 AM Viability Dye, ThermoFisher, Waltham, MA, USA) was added to 50 µL of diluted semen and incubated in the dark at room temperature for 10 min. Finally, 1.5 µL of 2.4 mM propidium iodide solution in water (LIVE/DEADTM Sperm Viability kit, ThermoFisher Scientific, Waltham, MA, USA) were added to 50 µL of diluted semen and incubated in the dark at room temperature for 7 min. For double staining (CaV + PI), PI was added to the sample following the minimum incubation time of CaV (10 min) and the sample was further incubated for 7 min. For triple staining (CaV + SYBR + PI), CaV and SYBR were added at the same time while PI was added 10 min later and the sample was further incubated for 7 min.

**Figure 1:**
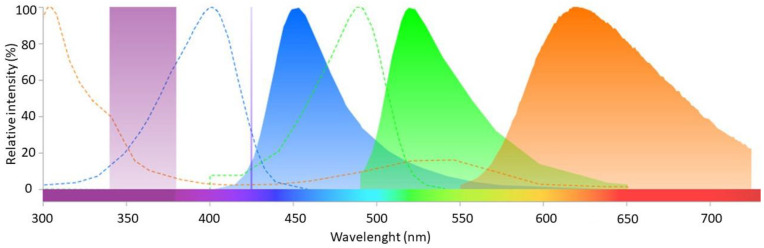
Fluorescence spectra of the three different fluorochromes employed: CaV, SYBR and PI. Dotted curves show the excitation spectra and plain curves the emission spectra. The violet plain column in the ultraviolet wavelengths represents the excitation filter employed, and the purple line at 425 nm represents the emission long pass filter. Light within the excitation filter is responsible for the excitation of the fluorochromes, and the emission filter transmits light of wavelength higher than 425 nm. The excitation (dotted) and emission (plain) graphs of CaV, SYBR and PI are illustrated in blue, green and orange, respectively. This figure was created with the online software Fluorescence SpectraViewer Chart from ThermoFisher Scientific, producer of the fluorochromes.

Fluorescent signals were visualised under a fluorescent microscope (Leica DM 2000 Led, Wetzlar, Germany) at final magnification X 630 (Leica ACS APO X 63, Wetzlar, Germany) in oil (ImmersolTM 518F, ThermoFischer, Waltham, MA, USA) with the Leica original cube filter A (Leica A 11513873, excitation 340–380 nm, dichroic 400 nm, emission LP425 nm, Wetzlar, Germany). The setting allowed direct observation of the spermatozoa and visualisation of the resultant emitted fluorescence from the 3 fluorochromes without creation of an overlay. The fluorescence spectra of Calcein Violet, SYBR^®^14 and propidium iodide (ThermoFisher) and the excitation and emission filters of this setting are illustrated in Figure 1. Images were taken with a colour digital microscope camera (Leica DFC450C, Wetzlar, Germany) and saved with the program Leica Application Suite (LAS Version 4.2.0, Wetzlar, Germany).

As detailed in Table 1, when SYBR and PI were used together, spermatozoa emitting green fluorescence were considered to have an uncompromised membrane integrity, while dead spermatozoa were countermarked in red by PI. With the dual CaV/PI stain, live and dead spermatozoa appeared in blue (head and tail) and red (head), respectively. Viability was calculated as the percentage of live cells and a minimum of 100 spermatozoa were evaluated. The complete set of dyes and observations was replicated twice on different days for all species.

#### 2.4.2. CaV Validation as a Marker of Acrosomal Membrane Integrity (Experiment 2)

Three ejaculates per species were evaluated. Each sample, after dilution (see Section 2.3), was divided in two. One sample was stained with CaV + SYBR + PI, and the second one was stained with CaV + Peanut agglutinin coupled to phycoerythrin (PE-PNA). Triple staining CaV + SYBR + PI was repeated as described above, allowing evaluation of membrane integrity. Double staining with CaV and PE-PNA was prepared using the same batch of CaV (eBioscienceTM Calcein Violet 450 AM Viability Dye, ThermoFisher, Waltham, MA, USA) and Peanut Lectin (GTXO1509, GeneTex, Alton Pkwy Irvine, CA, USA). A volume of 0.5 µL of a 1 mg/mL PE-PNA solution was added to 50 µL of diluted semen and incubated in the dark at room temperature for 30 min. Ten min before the end of incubation time of PE-PNA, a volume of 4 µL of a 93 µM CaV in PBS solution was added to the sample. Controls of single staining and unstained samples were also systematically prepared (SYBR, PI, CaV, PE-PNA, unstained). At the end of incubation period, 1.5 µL of ProlongTM Live Antifade Reagent (ThermoFisher, Waltham, MA, USA) was added to all samples regardless of the stain. 

As detailed in Table 1, spermatozoa emitting orange fluorescence (PE-PNA positive) from the acrosome part of the cell were considered to have a compromised acrosome.

The images of the spermatozoa were taken with an Observer 7 widefield fluorescent microscope (Zeiss, Germany), equipped with a 40 X/1.1 NA oil immersion objective. The samples were illuminated with a 385 nm, 475 nm, 555 nm and brightfield respectively for CaV, SYBR, PE and brightfield. Emission wavelength filters were 430–470 nm, 500–550 nm, 570–640 nm and none, respectively, for CaV, SYBR, PE-PNA and brightfield. Images were acquired using Axiocam 506 monochrome (Zeiss, Germany) and Zen Blue Program (Zeiss, Germany) automatically created overlay images. 

Blue fluorescence intensity emitted from the acrosome was measured as a relative value to the background blue auto-fluorescence using Zen Blue program (Zeiss, Germany) in triply stained samples. All samples were pooled by species. Intensity was compared between cells with both intact plasma membrane integrity and acrosome membrane (Mb+/Acr+), cells with impaired plasma membrane integrity and intact acrosome membrane (Mb-/Acr+), cells with intact plasm membrane integrity and impaired acrosome membrane (Mb+/Acr-) and, finally, cells with both impaired membrane and acrosome integrity (Mb-/Acr-).

### 2.5. Statistical Analysis

Statistical analysis was run on triply stained samples (SYBR/PI/CaV) and dual staining (CaV/PE-PNA) using Graphpad Prism^®^ (version 9.0 for Mac OSX, Graphpad Inc., San Diego, CA, USA) and statistical significance was established at *p* < 0.05.

The associations between Acrosome evaluation by CaV and PE-PNA, between plasma membrane integrity evaluation by CaV and SYBR/PI, and between plasma membrane integrity evaluated by SYBR/PI and acrosome membrane integrity evaluated by CaV, were all tested using an exact Fischer test.

## 3. Results

Parameters of routine semen evaluation evaluated prior to any staining or heat treatment are summarized in Table 2. Post heat treatment motility in all samples of all species was nil.

Observation of spermatozoa under brightfield and fluorescence microscopy was possible in all samples, and all species confirmed that fluorescence was emitted from a sperm cell and not from debris. In the negative control groups, where no dye was used, spermatozoa did not show any intrinsic fluorescence. Single stained and unstained samples were used as controls for cross positivity in fluorescence detection and analysed in each specific channel, confirming the absence of cross positivity and autofluorescence. Overall, heat-treated samples, used as dead controls, showed more than 90% of dead spermatozoa. These were marked in red when PI was used but did not show blue fluorescence within the head and the tail when CaV was used alone.

**Figure 2:**
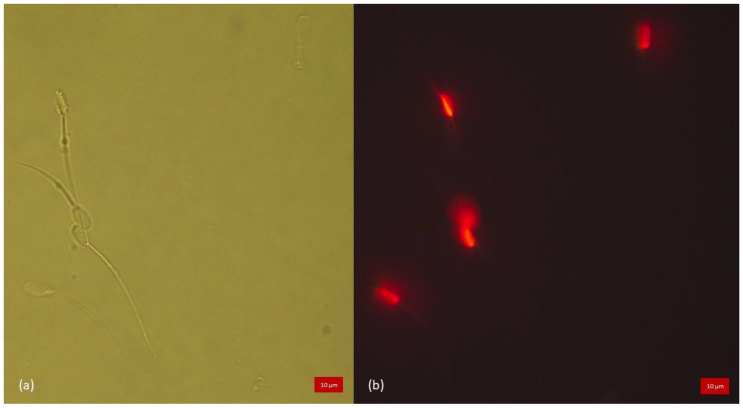
Heat-treated samples of boar semen triply stained with CaV, SYBR and PI. (**a**) Brightfield light microscopy allowing the localisation of all the spermatozoa. (**b**) Sample observed under fluorescence excitation light showing all spermatozoa positively marked for PI having lost their membrane integrity (resultant fluorescence, no overlay), confirming the efficacy of the heat treatment.

In all species, all samples, both heat-treated and not, displayed expected fluorescence of the tail and head when stained for positive (CaV) and/or negative (PI) marking of membrane integrity. In heat-treated samples, spermatozoa stained with PI fluoresced in red, demonstrating the loss of their membrane integrity, whereas those stained with CaV did not show blue fluorescence within the head or the tail, as illustrated in Figure 2. Percentages of sperm cells detected as alive per species are detailed in Table 2. There was a highly significant association for all species (*p* < 0.0001) between the evaluation by CaV and SYBR/PI used as reference dye.

**Figure 3:**
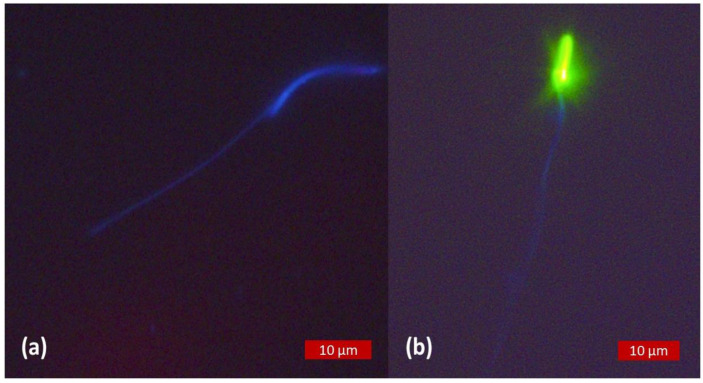
Non-heat-treated samples of rooster semen observed under fluorescence excitation light. Images obtained as direct observation of the resultant fluorescence, no overlay. (**a**) Dual staining with CaV and PI, the spermatozoa appear positively stained for membrane integrity with blue fluorescent head and tail. (**b**) Triple staining with CaV, SYBR and PI. The absence of fluorescence for PI staining indicates an intact plasma membrane. The live spermatozoon shows a blue tail, but the green fluorescence of SYBR resulting from our settings masks the blue fluorescence of CaV.

**Figure 4:**
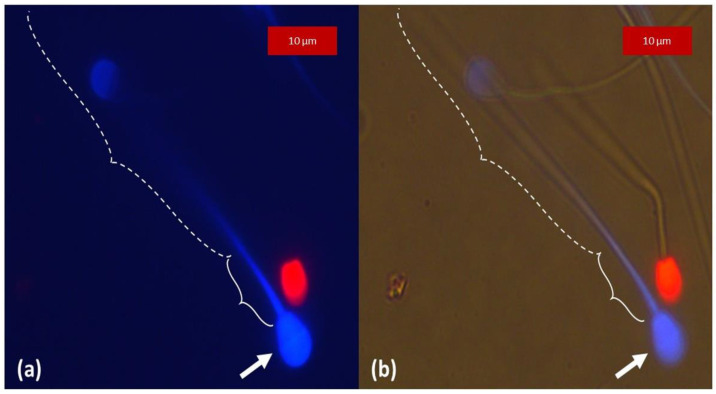
Non-heat-treated dog spermatozoa stained with CaV and PI. Images obtained as direct observation of the resultant fluorescence, no overlay. (**a**) Sample observed under fluorescence excitation light, the blue fluorescence of CaV was more intense in the head (arrow) and the first third of the tail, grossly corresponding to the midpiece (plain brackets), and progressively fading away towards the terminal end of the spermatozoa (dotted brackets). (**b**) Through fluorescence microscopy, the same sample was observed simultaneously under fluorescence excitation and brightfield light, allowing visualization of the tail and confirming the absence of labelling of its distal part (dotted brackets).

**Figure 5:**
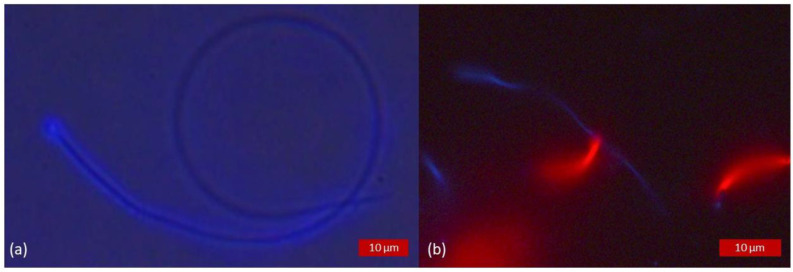
Non-heat-treated samples stained with CaV and PI. Images obtained as direct observation of the resultant fluorescence, no overlay. The limit between the head and the midpiece is not easily distinguishable in species with a small head. (**a**) Drone semen observed simultaneously under fluorescence excitation and brightfield light. (**b**) Rooster semen observed under fluorescence excitation light.

Subjectively, the intensity of the blue coloration of CaV seemed weaker and appeared to fade faster than the green and red ones of SYBR and PI, respectively. Sperm heads from samples with SYBR/CaV appeared green, as the more intense signal of SYBR produced with our settings masked the blue fluorescence of CaV in live spermatozoa, as shown in Figure 3. Whether used alone or in combination with PI, the blue fluorescence of CaV was more intense in the head and the first third of the tail, grossly corresponding to the midpiece, and progressively fading away towards the terminal end of the spermatozoa, which could not always be readily distinguished from the blue background, as detailed in Figure 4. In species with long and thin heads (rooster and drone) the limit between head and midpiece was not easily distinguished, as can be seen in Figure 5.

**Figure 6:**
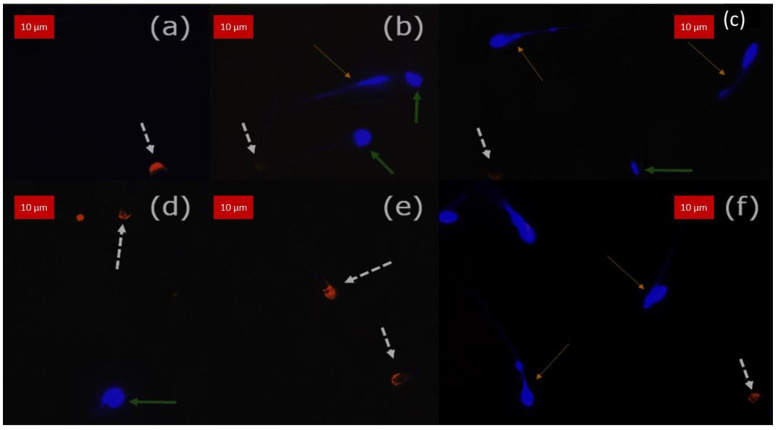
Double stained (CaV + PE-PNA) samples observed under fluorescence excitation light superposed to brightfield image of spermatozoa. Images obtained after overlay. The arrows point out three populations of spermatozoa. The white dotted arrows show reacted acrosomes with positive orange fluorescence. The green plain arrows point out spermatozoa with unreacted acrosomes but impaired membrane integrity. The thin yellow arrows point to sperm with intact acrosomes and intact membrane integrity. (**a**–**c**) Bull semen, (**d**–**f**) Stallion semen. Slight drifting may be observed between the brightfield and the blue channel (**b**–**e**).

**Figure 7:**
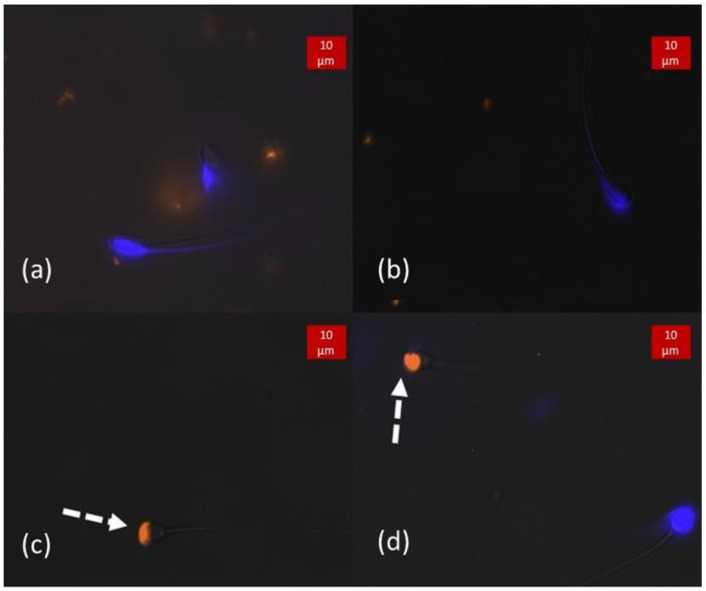
Double stained (CaV + PE-PNA) samples observed under fluorescence excitation light. (**a**,**b**) Boar semen showing no orange fluorescence, demonstrating the integrity of its acrosomes. (**b**) Observation of the acrosome with bright blue fluorescence corresponding with the acrosomal region of the spermatozoa. (**c**,**d**) Dog semen with reacted spermatozoa, as marked by PE-PNA (pointed out by dotted arrows). (**d**) On the right-hand side, no fluorescence of the tail of the sperm is observed, inferring that the membrane integrity is lost. However, bright blue fluorescence is still present on the head while no orange fluorescence is emitted, thus the acrosome is intact.

As illustrated in Figure 6 and Figure 7, samples stained with CaV and PE-PNA showed that reacted acrosome were stained in orange, and unreacted acrosomes were stained in blue regardless of cell membrane integrity, showing them to have intact acrosome membranes. Comparison of percentage of intact acrosome evaluated by CaV and by PE-PNA, the reference dye, is presented in Table 3. No spermatozoa appeared positive for CaV and PE-PNA at the same time. There was a very significant association between the evaluation of acrosomal integrity by CaV and by PE-PNA (*p* < 0.0001 for boar, stallion and bull and *p* = 0.0003 for dog).

**Figure 8:**
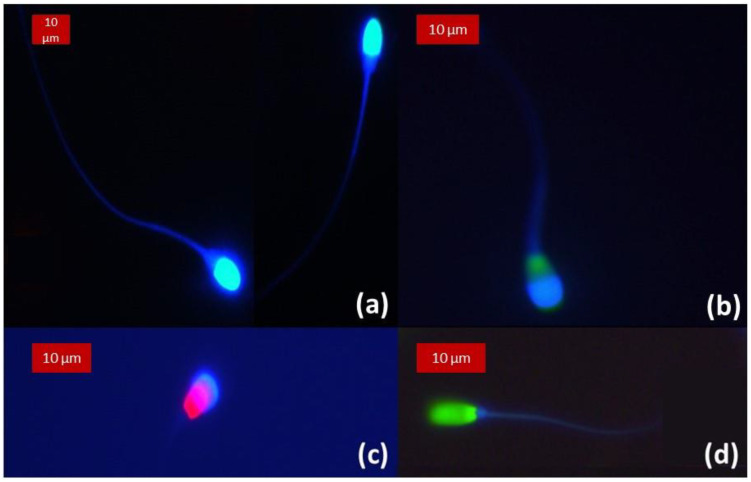
Samples observed under fluorescence excitation light, no overlay. (**a**) Stallion semen stained with CaV and PI. Superposition of the blue fluorescence from the head and the acrosome is observable with high brightness at the apex of the head. (**b**) Bull semen triply stained with CaV, SYBR and PI. The apex of the head fluoresces in blue, marking the intact acrosome. It superposes to the green fluorescence of SYBR marking the nucleus. (**c**) Dog semen with dual staining (CaV and PI) showing a spermatozoon with an intact acrosomal membrane but impaired membrane integrity. (**d**) Boar semen triply stained with CaV, SYBR and PI displaying cytoplasmic membrane integrity. The absence of blue fluorescence at the apex of the head indicates the impairment of the acrosome.

**Figure 9:**
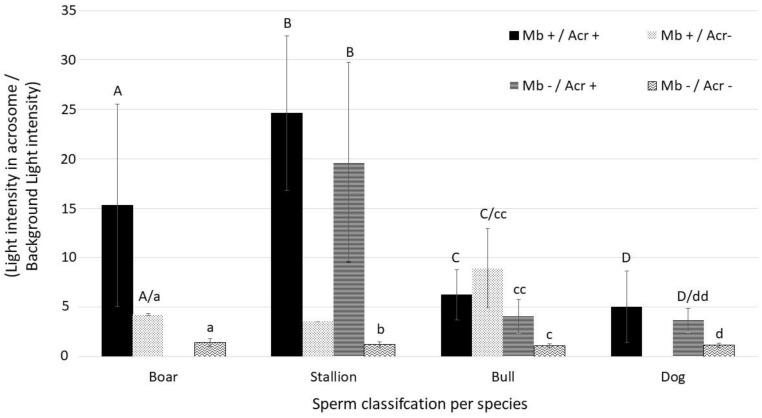
Summary of relative intensity of acrosome per species. Intra-species/inter sperm class statistical analysis. Different letters indicate a statistical difference (*p* < 0.05).

As illustrated in Figure 8, in samples stained with CaV alone or in combination with SYBR and or PI, irrespectively from cell membrane integrity, the unreacted acrosomes were stained in blue and distinguishable from the heads in species with large and round heads. This was not observed in spermatozoa from roosters and drones whose heads are comparatively small. Generally, blue fluorescence intensity from intact acrosome was subjectively higher than the other parts of the spermatozoa. Number of spermatozoa with intact acrosome membrane are detailed in Table 3 and those with intact plasma membrane in Table 4. There was a highly significant association (*p* < 0.0001) between membrane integrity (Mb+) evaluated by the reference dye (SYBR/PI) and acrosome membrane integrity (Acr+) evaluated by CaV in species where the acrosome could be evaluated (i.e., boar, bull, stallion and dog). Figure 9 details the relative fluorescence intensity measured from the acrosomes in the four sperm cell classes. It was significantly higher in Mb+/Acr+ compared to Mb-/Acr- for all species evaluated (*p* < 0.001). Interestingly and surprisingly, a significant difference in fluorescence intensity measured from the acrosome was found between Mb+/Acr+ and Mb-/Acr+ for the bull (*p* < 0.05) and a tendency for the dog (*p* = 0.056). No spermatozoa with Mb+/Acr- could be found in dog and in only one stallion sample, and very low numbers could be found in the boar and bull samples. No spermatozoa with Mb-/Acr+ were found in boar samples. In our samples, more than 30% of stallion sperm cells had an impaired plasma membrane with an intact acrosome membrane (Mb-/Acr+).

## 4. Discussion

Objective validation of a dye requires individual examination of the marked cells, precluding the use of automated sperm cells counting, such as fluorescence-based CASA or flow cytometry [29], and was thus performed manually by one experienced operator in our study. Semi-automatic assessment of viability on photographs can be achieved on, non-motile, cell monolayers [46]. However, the low number of cells per image required for individual evaluation, their crossing in and out of the frame and the relatively rapid bleaching of the fluorescence [47] completely limit this approach for semen samples. Epifluorescence was therefore used in our study to validate CaV for semen evaluation. The movements of the targeted cells preclude manual change of filters during examination to adjust to the most adequate wavelength of the different fluorochromes and, later, superposition of serial pictures, as commonly performed in epifluorescence. Acrosomal status was therefore evaluated using a microscope equipped with an automatic filter changing system, allowing instant superposition of signals and creation of a resulting superposed image. Even using this technique, drifting may be observed in some samples (Figure 7). Fluorescence bleaching was subjectively found to be faster for CaV than for SYBR and PI, this may be due to a higher DNA than esterases concentration and a subsequent higher fluorescence emission, but also because of the technical assembly of the filter cube. The addition of antifade product did not significantly modify this higher bleaching observation. This could also be explained by the characteristics of the CaV itself as it fluoresces in blue, and photobleaching has been shown to be higher for lower wavelengths due to their intrinsic higher energy production [48]. Its shorter lifespan makes it a better candidate for further development with flow cytometry, which allows rapid analysis of numerous events before fluorescence bleaching occurs in comparison with individual observation under fluorescence microscopy, which takes longer. 

Surprisingly, during evaluation of membrane integrity in Experiment 1, we observed emitted fluorescence by SYBR^®^14, even when used alone. As shown in Figure 1, the technical assembly should have prevented excitation of SYBR^®^14, as no wavelength above 380 nm should reach the sample, and yet SYBR^®^14, which is normally excited by wavelengths 400 nm and above, showed fluorescence. This may probably be explained by the low specificity of excitation wavelengths of the SYBR fluorochromes family. Interestingly, the fluorescence spectrum provided by the manufacturer for SYBR^TM^ Green I (ThermoFisher Scientific, Waltham, MA, USA), another fluorochrome from the same family whose fluorescence is supposed to be similar to that of SYBR^®^14, shows two supplementary low excitation peaks at 300 nm and 375 nm [49]. It may be suspected that SYBR^®^14 displays a yet undocumented similar excitation pattern as SYBR^TM^ Green I, which explains the unexpected excitation of SYBR^®^14 we observed.

Motility has been shown to correlate with viability in equine semen, and a similar relationship has been reported in bovine andrology [50,51,52]. This study did not aim at establishing such an association or correlation between motility and viability, which would not have been possible because of the low number of samples. However, this reported correlation was used as a gross expectation for viability. When no motility was observed in heat-treated samples, it was expected to have no or almost no spermatozoa with intact membrane, which was confirmed by our results with PI fluorochrome as reported in Table 3. PI staining is an established method to mark non-viable sperm cells in different species [53]. In our dead control group, all heat-treated spermatozoa were PI positive and identified as having lost their membrane integrity. The same results were obtained in all species and agreed with the results obtained after CaV staining. To the best of our knowledge, this is the first report of validation of CaV that included a control group to evaluate the labelling of sperm cells that have been heat-treated. Viability rates calculated either by SYBR/PI or CaV (head and tail) were highly similar and statistically associated in all species (*p* < 0.0001) confirming the efficacy of CaV to evaluate sperm cell integrity. Although recently mentioned in the literature for bovine semen evaluation [3,5,17,54,55], the use of CaV for that purpose appears to have never been validated. Using heat-treated samples as dead controls and PI as an established marker of viability, our results demonstrate that CaV can be used as a positive membrane integrity marker to include in semen evaluation of horses, bulls, dogs, boars, roosters and drones.

Esterases have been described to be generally distributed in the cell [18,19]. Intensity of blue fluorescence seemed to decrease from the head to the tail of the spermatozoa in species with large heads, unlike drone and rooster. These results suggest that esterases are equally distributed in the cytoplasm whose volume decreases from the head to the tail. For rooster and drone samples, because of the very small size of the head, the difference in intensity between the head and the midpiece could not be observed as both segments have a similar volume of cytoplasm. On the contrary, the second half of the tail was clearly less fluorescent. This supports the likely homogenous distribution of esterases within the cytoplasm in these species too. In samples from all species where the size of the sperm head allowed detailed observation, unreacted acrosomes showed intense blue fluorescence with or without positive marking of the rest of the cell. The small size of the heads of drones and roosters’ spermatozoa prevented a similar observation in our conditions. However, as esterases have been shown to be present in the acrosome in other species [19,20,22], it may be suspected that acrosomal status of drones and roosters could also be assessed by CaV, using higher magnification observation to locate precisely the origin of the blue signal within the small head. In order to confirm the labelling of the unreacted acrosome by CaV, these results were compared to those of PE-PNA, a validated marker of the acrosome reaction [24,25,26,27]. Binding properties of PE-PNA are not clearly reported for rooster spermatozoa [56], and to the best of the authors’ knowledge, they have also not been reported for drone semen. These two species were therefore not included in this part of the experiment. In our other species of interest, PE-PNA positive spermatozoa were considered having impaired acrosome membrane integrity regardless of its underlying cause (activation or impaired cytoplasmic membrane integrity). The PE-PNA positive population was used as control for CaV, as mutual exclusion of fluorescence between PE-PNA and CaV was expected based on their intrinsic biochemical mechanism. No spermatozoa with simultaneous acrosomal orange (PE-PNA+) and blue (CaV+) fluorescence were observed. Evaluation of the acrosomal status by PE-PNA and CaV shows a statistically significant association between the two techniques, confirming that CaV can be used as an interesting a marker for acrosome membrane integrity in horses, bulls, dogs and boars and potentially for roosters and drones by epifluorescence.

Because esterases are present in both the cytoplasm and the acrosome, the intensity of fluorescence of live spermatozoa with unreacted acrosome (Mb+/Acr+) could be expected to be higher than that with reacted acrosome (Mb+/Acr-), thus allowing differentiation between these two subpopulations of live spermatozoa. However, this subpopulation of live spermatozoa with a reacted acrosome (Mb+/Acr-) is of rare natural occurrence [57], which was also observed in our study. As rapid bleaching limits the possibility of observing such sperm cells under epifluorescence, we suggest that flow cytometry, where thousands of events may be rapidly evaluated, will help to identify this subpopulation in the future. Based on its very function, we speculated that the acrosome would be particularly rich in esterases, as already suggested in the literature [20]. Therefore, it was expected that the relatively minimal difference of fluorescence associated with an impaired cytoplasmic membrane would not allow the difference between Mb+/Acr+ and Mb-/Acr+ cells. This is what we clearly observed in dogs and stallions. Surprisingly, this was not the case for the bulls. However, this result should be taken with caution and be confirmed in other studies that include more samples and techniques allowing precise localization of the esterases, as the current literature about esterase activity in the sperm cell is very limited.

Motility is a predictive parameter of fertility in domestic animals [7,8,9,10,11,12] but cannot explain low fertility on its own. Semen evaluation must be run on multiple parameters, such as viability, acrosomal status, DNA fragmentation and lipid peroxidation, to develop reliable predictive models of fertility [2,6]. Multiplying the number of parameters evaluated per semen sample by repeating the analyses seems easy for species with large volume ejaculates. However, it is time consuming and self-limiting for species and donors with small ejaculated volumes, such as roosters, some small dogs and even worse for drones, with approximately 1 µL collected per individual [58], and simultaneous multiple parameter analysis surely is an answer to this. An obvious limitation to fluorescent analysis is spectrum overlapping. For instance, routinely used fluorochromes for lipid peroxidation and DNA fragmentation emit in green and red light spectrum channels [16], limiting their possible combinations with SYBR/PI as only one single fluorochrome emitting in one channel can be used at a time. Thus, the use of blue fluorescence to evaluate viability while freeing other fluorescent channels has been suggested [16]. We suggest here that further work is needed for technical adjustments and thorough validation of CaV for flow cytometry. CaV could then be used to evaluate both viability and acrosomal status in association with PI, or other dyes, leaving the green spectrum channels free to assess other semen characteristics in a multiple parameters analysis by flow cytometry if the subpopulation Mb+/Acr- is identified. Indeed, flow cytometry multiple parameter analysis outperforms its epifluorescent counterpart in terms of numbers of evaluated events and time dedicated to the analysis.

## 5. Conclusions

CaV fluoresces in blue, which frees the green channel spectrum or both the green and red light spectrum channels. This broadens the possibilities of combinations between fluorochromes to expand the range of parameters simultaneously evaluated in a multiple parameters analysis of semen. This is obviously particularly beneficial for species or donors with small volume of ejaculate, such as drones, and for wildlife animals where semen collection is challenging and usually requires general anaesthesia.

Although its use had previously been suggested for that purpose, this is the first report that thoroughly tests and validates CaV as a marker of sperm membrane integrity and thus viability. Our results also demonstrate for the first time that CaV can be used to assess acrosomal integrity in horses, bulls, dogs, boars. Establishing CaV as a marker of membrane integrity through fluorescence microscopy is a milestone that leads the way to its further study and application with flow cytometry.

## Figures and Tables

**Table 1:** Sperm classification by assessment of plasma and acrosomal membrane integrity.

	PI Nucleus (RED)	SYBR Nucleus (GREEN)	CaV Cytoplasm (BLUE)	PE-PNAacrosome (ORANGE)	CaV Acrosome (BLUE)
Intact plasma membrane/intact acrosome (Mb+/Acr+)	-	+	+	-	+
Intact plasma membrane/damaged acrosome (Mb+/Acr-)	-	+	+	+	-
Damaged plasma membrane/intact acrosome (Mb-/Acr+)	+	-	-	-	+
Damaged plasma membrane/damaged acrosome (Mb-/Acr-)	+	-	-	+	-

PI = propidium iodide, membrane impermeant marks the nucleus of damaged plasma membrane cells in red, SYBR = SYBR14, membrane permeant marks the nucleus of all cells in green, CaV = Calcein Violet membrane permeant marks cytoplasm and acrosome of cells with intact plasma membrane and/or intact acrosome in blue, PE-PNA = phycoerythrin conjugated, membrane impermeant marks damaged acrosome in orange; + = positive fluorescence, - = absence of fluorescence.

**Table 2:** Summary of semen evaluation. Mean results of the replicates are presented.

Species	Motility (%)	Progressive Motility (%)	Concentration (×10^6^/mL)
Boar	84 **^±^**^12^	49 **^±^**^12^	184.1 **^±^**^32^
Bull	39 **^±^**^9^	29 **^±^**^10^	87 **^±^**^47.9^
Dog	95 **^±^**^0^	87 **^±^**^3^	623.6 **^±^**^210.8^
Stallion	22 **^±^**^17^	16 **^±^**^15^	177 **^±^**^114^
Drone	25 **^±^**^0^	Nonevaluated	4800 ^±3394.1^
Rooster	68 **^±^**^38^	28 **^±^**^4^	214 **^±^**^192.3^

**Table 3:** Percentage of intact acrosome.

Species	PE-PNA -	CaV +
Boar	89.9 ^±7.2^	89.9 ^±7.2^ *
Bull	74 ^±8.1^	68.7 ^±17.2^ *
Dog	87.5 ^±21.7^	87.5 ^±21.7^ *
Stallion	84.7 ^±5.1^	71.8 ^±17.4^ *

* significant association of CaV result to PE-PNA results.

**Table 4:** Percentage of intact plasma membrane (Live spermatozoa).

Non-Heat-Treated Samples			Heat-Treated Samples		
Species	SYBR+ PI-	CaV +	Species	SYBR+ PI-	CaV +
Boar	62.4 ^±11.4^	62.4 ^±11.4^ *	Boar	0 ^±0^	0 ^±0^
Bull	51.7 ^±12/0.2^	48.9 ^±9.4^ *	Bull	0.6 ^±1.1^	0 ^±0^
Dog	73.8 ^±5.5^	72.4 ^±4.8^ *	Dog	0 ^±0^	0 ^±0^
Stallion	30.2 ^±20.9^	29.8 ^±21^ *	Stallion	0 ^±0^	0 ^±0^
Drone	66.3 ^±5.7^	66.3 ^±5.7^ *	Drone	6.5 ^±0.7^	0 ^±0^
Rooster	63.6 ^±7.4^	63.6 ^±7.4^ *	Rooster	9.5 ^±0.7^	0 ^±0^

* significative association of CaV result to SYBR/PI results.

## Data Availability

Data is contained within the article.
